# A Disproportionality Analysis of Immune Checkpoint Inhibitors in Combination With Platinum‐Based Agents Using the FDA Adverse Event Reporting System Database

**DOI:** 10.1002/cam4.71527

**Published:** 2026-03-30

**Authors:** Boyi Liu, Wenchao Zhang, Ruizhe Huang, Dinwen Liu, Jiaxing Liu, Ao Han, Yike Li, Danna Chen

**Affiliations:** ^1^ The First Clinical College Changsha Medical University Changsha China; ^2^ Institute of Cytology and Genetics, Basic Medical School University of South China Hengyang China; ^3^ Changsha Hospital for Maternal & Child Health Care Changsha China; ^4^ Department of Basic Medical Sciences Changsha Medical University Changsha Hunan China; ^5^ Hunan Provincial University Key Laboratory of the Fundamental and Clinical Research on Functional Nucleic Acid Changsha China

**Keywords:** disproportionality analysis, FAERS, immune checkpoint inhibitors, platinum‐based agents

## Abstract

**Objective:**

Immune checkpoint inhibitors (ICIs) combined with platinum‐based compounds are commonly used in the treatment of certain malignant tumors. This study aims to analyze adverse events (AEs) associated with the combination therapy of ICIs and platinum‐based compounds by using the FAERS database.

**Methods:**

This study retrieved relevant adverse event (AE) data from the FAERS database (2008–2024) and conducted a retrospective analysis of the collected AEs. Multiple disproportionality analysis algorithms were employed, including the Reporting Odds Ratio (ROR), Proportional Reporting Ratio (PRR), Bayesian Confidence Propagation Neural Network (BCPNN), and Multi‐item Gamma Poisson Shrinker (MGPS).

**Results:**

Analysis of 28,585 reports identified 27 significant SOC‐level signals, strongest for hematological (ROR = 6.3), endocrine (ROR = 14.3), and hepatobiliary disorders (ROR = 4.49). Key PTs included malignant neoplasm progression (ROR = 16), febrile neutropenia (ROR = 15.31), and myocarditis (ROR = 16.3). 45.5% of AEs occurred within 1 month (median onset: 38 days). Combination therapy showed lower rates vs. monotherapy for malignancy progression and nephrotoxicity, but higher neurotoxicity (628 neuropathy cases).

**Conclusion:**

The combination exhibits distinct early‐onset toxicities (early‐onset hematological/endocrine/hepatic) and novel risks (neuropathy/myocarditis/leukemia), necessitating enhanced initial monitoring and subgroup‐specific management.

## 1 Introduction

Primary bronchogenic carcinoma, commonly referred to as lung cancer (LC), is primarily classified into non‐small cell lung cancer (NSCLC) and small cell lung cancer (SCLC) [1]. As one of the most prevalent malignant tumors worldwide, it represents the leading cause of cancer‐related mortality [2, 3]. NSCLC accounts for approximately 85% of all lung cancer cases [4], with treatment modalities including surgery, chemotherapy, radiotherapy, targeted therapy, and immunotherapy [5]. In recent years, immune checkpoint inhibitors (ICIs) (such as PD‐1/PD‐L1 inhibitors) have played an increasingly important role in the treatment of advanced NSCLC [6]. SCLC constitutes about 15% of lung cancer cases, and its therapeutic approach is primarily determined by disease stage (limited‐stage vs. extensive‐stage) [7], with chemotherapy remaining the mainstay treatment for extensive‐stage SCLC (ES‐SCLC) [8].

With the rapid development of tumor immunology, ICIs have achieved remarkable progress in the treatment of malignant tumors [9]. However, studies indicate that ICIs may induce adverse reactions including endocrine toxicity, cutaneous toxicity, and gastrointestinal toxicity [10, 11, 12], while platinum‐based chemotherapeutic agents are commonly associated with side effects such as gastrointestinal toxicity and nephrotoxicity [13, 14]. A systematic review indicated that treatment regimens involving camrelizumab plus chemotherapy or avelumab plus chemotherapy were associated with a significantly increased incidence of adverse events (AEs) of any grade. In contrast, therapies combining ipilimumab, durvalumab, or pembrolizumab with chemotherapy demonstrated a comparatively safer profile [15].

One FAERS study indicated that colitis, pneumonia, and interstitial lung disease are common immune‐related AEs associated with ICI therapy [16], while another FAERS study reported that adverse reactions related to platinum‐based compounds primarily involved blood and lymphatic system disorders, gastrointestinal diseases, and respiratory system disorders [17]. Therefore, in‐depth analysis and evaluation of the adverse reactions resulting from this combination therapy are of significant clinical importance.

The FDA Adverse Event Reporting System (FAERS), established by the U.S. Food and Drug Administration (FDA), is a pivotal pharmacovigilance database that collects and analyzes spontaneous reports of AEs from worldwide sources. As the largest global drug safety surveillance system, it provides critical support for post‐market drug monitoring and risk identification. However, when interpreting findings derived from FAERS, several important limitations must be considered. These include underreporting of events, the lack of a definitive denominator (i.e., the exact number of patients exposed to a drug), and potential reporting biases. Therefore, while FAERS is highly valuable for detecting potential safety signals and generating hypotheses, its results should be interpreted with caution and validated through further controlled prospective studies.

This study systematically compared the adverse event (AE) profiles of three treatment regimens using FAERS database: ICI monotherapy, platinum‐based compound monotherapy, and their combination therapy. Through comprehensive evaluation of reported AEs, it aims to provide real‐world evidence for the clinical safety profile of ICIs combined with platinum‐based compounds.

## 2 Materials and Methods

### 2.1 Study Design and Data Sources

This study extracted adverse event (AEs) data from the FAERS database spanning the first quarter of 2008 to the fourth quarter of 2024, with a specific focus on reports involving combination therapies of five ICIs (durvalumab, pembrolizumab, ipilimumab, atezolizumab, nivolumab) and three platinum‐based compounds (cisplatin, carboplatin, oxaliplatin).

The data for this study were obtained from the FDA Adverse Event Reporting System (FAERS) maintained by the U.S. Food and Drug Administration (FDA). This publicly accessible pharmacovigilance database aggregates treatment‐related adverse event (AE) reports worldwide, serving as a critical tool for drug safety signal detection and risk assessment.

### 2.2 Data Extraction

The FAERS database comprises seven primary data tables: DEMO (patient demographic and administrative information), DRUG (drug information), REAC (adverse event reports), OUTC (patient outcomes), RPSR (report source information), INDI (treatment indications), and THER (start/end dates of reported drug therapies). The DRUG table specifically documents four drug role types: PS (primary suspect drug), SS (secondary suspect drug), C (concomitant drug), and I (interacting drug).

Serious patient outcomes are defined by the FDA as: DE (death), LT (life‐threatening), HO (initial or prolonged hospitalization), DS (disability), CA (congenital anomaly), and OT (other serious medical events). In this study, case identification was performed through drug name queries in the DRUG table, with subsequent screening limited to PS cases.

All AEs were standardized using the Medical Dictionary for Regulatory Activities (MedDRA version 27.0), which features a five‐tier hierarchical structure: SOC (System Organ Class), HLGT (High Level Group Term), HLT (High Level Term), PT (Preferred Term), and LLT (Lowest Level Term).

### 2.3 Data Cleaning

**FIGURE 1:**
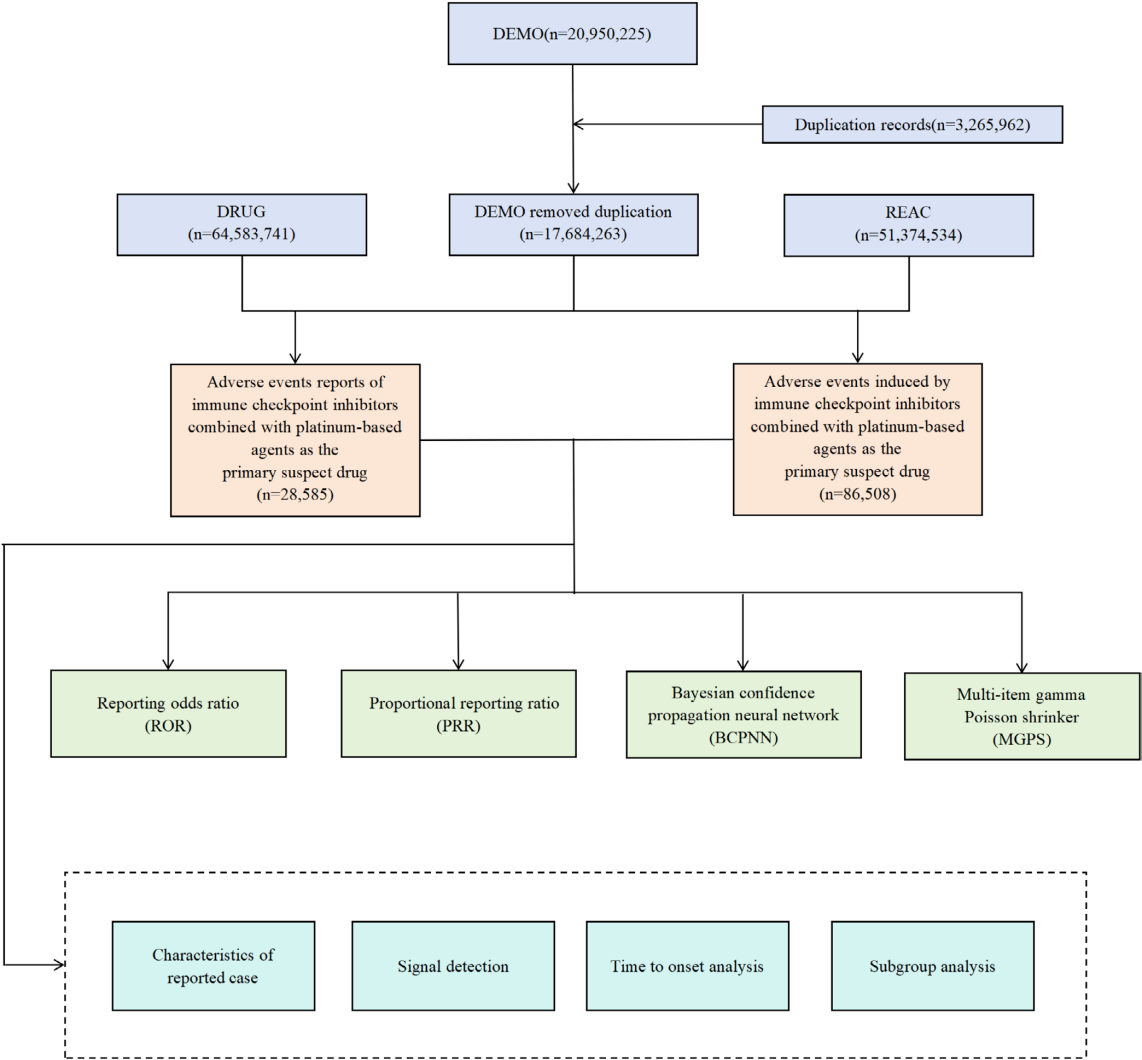
Flowchart illustrating the screening process for adverse events (AEs) associated with combination therapy of immune checkpoint inhibitors and platinum‐based compounds from the FAERS database. Duplicates were removed using the FDA‐recommended deduplication logic. DEMO, Patient demographic and administrative information; DRUG, Drug information; REAC, Adverse event reports.

In this study, we employed the FDA‐recommended methodology to retrieve all reports related to the combination therapy of ICIs and platinum‐based compounds from January 2008 to December 2024. To address the inevitable presence of duplicate reports in the FAERS database and ensure data accuracy and authenticity, we applied the FDA‐recommended deduplication method. The procedure was performed as follows: The fields PRIMARYID, CASEID, and FDA_DT were selected from the DEMO table. Records were sorted in ascending order by CASEID, FDA_DT, and PRIMARYID. For reports sharing the same CASEID, the entry with the latest FDA_DT was retained. In cases where both CASEID and FDA_DT were identical, the record with the highest PRIMARYID value was kept. The detailed data processing workflow is illustrated in Figure 1.

### 2.4 Disproportionality Analysis

Disproportionality analysis serves as a fundamental methodology in pharmacovigilance and drug safety monitoring. In this study, we employed four analytical approaches: Reporting Odds Ratio (ROR), Proportional Reporting Ratio (PRR), Bayesian Confidence Propagation Neural Network (BCPNN), and Multi‐item Gamma Poisson Shrinker (MGPS). Higher values of ROR, PRR, BCPNN, and MGPS indicate stronger adverse event (AE) signals and more significant statistical associations between the target drug and target AE. The mathematical formulas and threshold criteria for all four algorithms are detailed in Table S1. All analyses were performed using R software (version 4.2.3).

### 2.5 Subgroup Analysis

Subgroup analysis serves as a critical methodological approach in pharmacovigilance research. This study employed subgroup analysis to evaluate heterogeneity in drug effects and safety profiles. Stratified analyses were conducted based on predetermined demographic characteristics (age, gender) to identify high‐risk populations, validate result consistency, and explore potential Preferred Term (PT) variations.

### 2.6 Time‐to‐Onset Analysis

We initially excluded entries with erroneous or incomplete date information, as well as AEs reported prior to treatment initiation. Subsequently, the time‐to‐onset interval was calculated based on the temporal difference between EVENT_DT (adverse event occurrence date) and START_DT (treatment initiation date).

### 2.7 Data Visualization

All statistical graphics were generated using R software (version 4.2.3) with the ggplot2 package, supplemented by WPS Office (version 12.1) for final figure preparation.

### 2.8 Ethical Statement

The data from the FAERS database is freely available for download and use. This study does not require ethical review or approval but must comply with relevant laws and regulations.

## 3 Results

### 3.1 Descriptive Analysis

**FIGURE 2:**
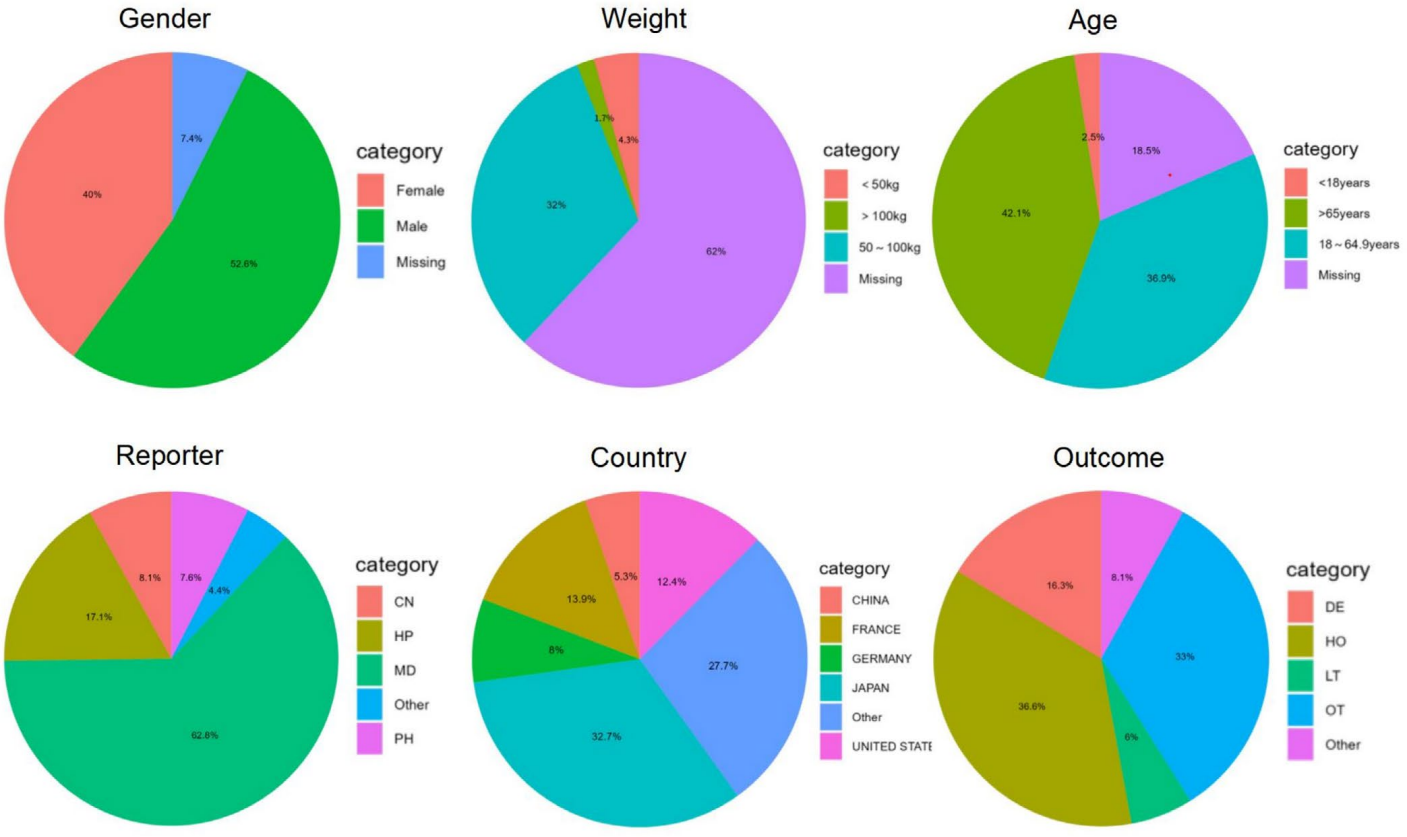
Clinical features of immune checkpoint inhibitors combined with platinum compounds reported in the FAERS database.

**FIGURE 3:**
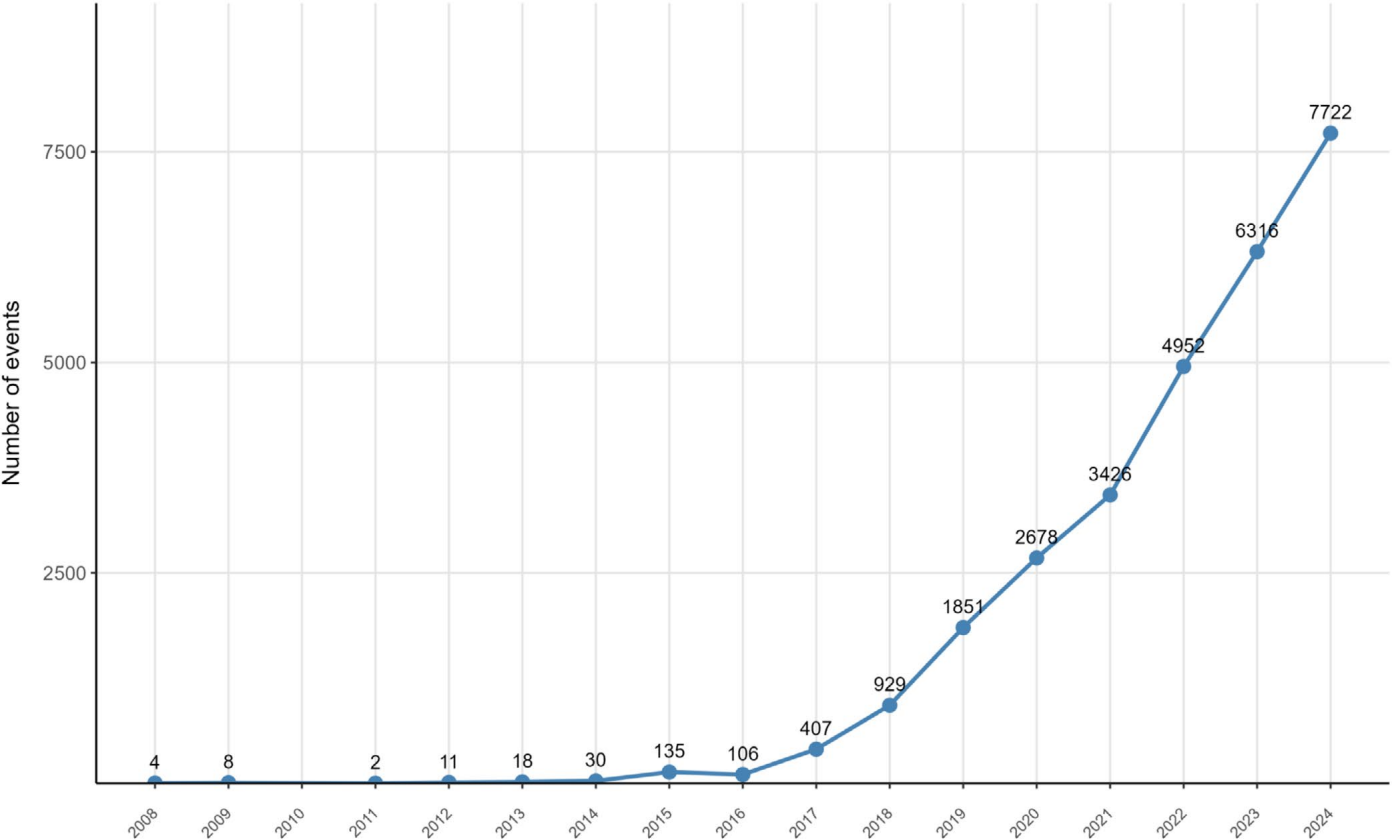
Annual distribution of adverse event reports associated with immune checkpoint inhibitors combined with platinum compounds from 2008 to 2024.

A total of 20,950,225 adverse event reports were extracted from the FAERS database spanning the first quarter of 2008 to the fourth quarter of 2024, among which 28,585 reports were associated with combination therapy of ICIs and platinum‐based compounds. Figure 2 presents the demographic and clinical characteristics of these reports, including patient age, sex, body weight, reporting country, source of report, and outcomes. The sex distribution showed 40% female and 52.6% male patients. Among reports with specified geographical data, the top three reporting regions were Japan (32.7%), France (13.9%), and the United States (12.4%). Age stratification revealed that 36.9% of patients were aged 18–65 years, 42.1% were over 65 years, while only 2.5% were under 18 years. Weight distribution indicated the highest proportion in the 50–100 kg range (32%), followed by patients weighing < 50 kg (4.3%). The majority of reports were submitted by healthcare professionals (MD, 62.8%), with the most frequent outcomes being hospitalization (HO, 36.6%) and death (DE, 16.3%). Figure 3 demonstrates a rapid annual increase in adverse event reports since 2016, reaching 7722 cases in 2024. Detailed clinical characteristics are provided in Table S2.

### 3.2 Signal Detection Based on SOC Levels

We systematically evaluated the System Organ Class (SOC) level signals associated with the combination therapy of ICIs and platinum‐based compounds. Among the 27 SOCs identified as significant in our analysis, we further classified them into two categories: (1) SOCs already documented in the prescribing information of ICIs or platinum‐based agents, and (2) potential SOCs not currently highlighted in the product labels but detected as significant signals in our FAERS analysis.

As shown in Table 1, several SOCs with strong signal scores aligned with known toxicities described in the product labels. These included:

① Blood and lymphatic system disorders (ROR = 6.3, PRR = 5.78, EBGM = 5.73, IC = 2.52), consistent with the myelosuppressive effects of platinum‐based agents.
② Endocrine disorders (ROR = 14.3, PRR = 13.84, EBGM = 13.57, IC = 3.76), a well‐established class effect of immune checkpoint inhibitors.
③ Hepatobiliary disorders (ROR = 4.49, PRR = 4.36, EBGM = 4.33, IC = 2.12), also recognized in the labeling of both drug classes.

**TABLE 1:** Signal strength at the System Organ Class level for adverse event reports involving combination therapy of immune checkpoint inhibitors with platinum‐based compounds in the FAERS database.

SOC	Case reports	ROR (95% Cl)	PRR (*χ* ^2^)	EBGM (EBGM05)	IC (IC025)
General disorders and administration site conditions	9942	0.61 (0.6–0.63)	0.66 (2146.68)	0.66 (0.65)	−0.6 (−0.63)
Gastrointestinal disorders	8509	1.16 (1.14–1.19)	1.15 (177.41)	1.15 (1.13)	0.2 (0.17)
Blood and lymphatic system disorders	8490	6.3 (6.16–6.44)	5.78 (33799.54)	5.73 (5.63)	2.52 (2.49)
Investigations	7038	1.34 (1.31–1.37)	1.31 (553.25)	1.31 (1.28)	0.39 (0.35)
Respiratory, thoracic and mediastinal disorders	6164	1.53 (1.49–1.57)	1.49 (1036.51)	1.49 (1.46)	0.57 (0.53)
Infections and infestations	5997	1.33 (1.3–1.37)	1.31 (459.91)	1.31 (1.28)	0.39 (0.35)
Skin and subcutaneous tissue disorders	4769	1.02 (0.99–1.05)	1.02 (1.9)	1.02 (0.99)	0.03 (−0.01)
Nervous system disorders	4606	0.61 (0.59–0.62)	0.63 (1114.28)	0.63 (0.61)	−0.67 (−0.72)
Neoplasms benign, malignant and unspecified (incl cysts and polyps)	4395	1.97 (1.91–2.03)	1.92 (1993.37)	1.92 (1.87)	0.94 (0.9)
Injury, poisoning and procedural complications	4013	0.46 (0.45–0.48)	0.49 (2356.34)	0.49 (0.48)	−1.03 (−1.08)
Metabolism and nutrition disorders	3457	1.89 (1.83–1.96)	1.86 (1396.17)	1.86 (1.8)	0.89 (0.84)
Hepatobiliary disorders	3439	4.49 (4.34–4.65)	4.36 (8911.49)	4.33 (4.21)	2.12 (2.06)
Renal and urinary disorders	3272	2.1 (2.02–2.17)	2.06 (1800.7)	2.05 (1.99)	1.04 (0.99)
Endocrine disorders	3003	14.3 (13.79–14.84)	13.84 (35097.55)	13.57 (13.15)	3.76 (3.71)
Musculoskeletal and connective tissue disorders	2297	0.49 (0.47–0.51)	0.5 (1202.3)	0.5 (0.48)	−1 (−1.06)
Cardiac disorders	2242	0.98 (0.94–1.02)	0.98 (1.06)	0.98 (0.95)	−0.03 (−0.09)
Vascular disorders	1565	0.83 (0.79–0.88)	0.84 (50.95)	0.84 (0.8)	−0.26 (−0.33)
Immune system disorders	1080	1.12 (1.05–1.19)	1.12 (13.48)	1.12 (1.06)	0.16 (0.07)
Psychiatric disorders	743	0.14 (0.13–0.15)	0.15 (3771.52)	0.15 (0.14)	−2.73 (−2.83)
Eye disorders	540	0.31 (0.28–0.33)	0.31 (848.12)	0.31 (0.29)	−1.69 (−1.81)
Surgical and medical procedures	437	0.37 (0.33–0.4)	0.37 (474.81)	0.37 (0.34)	−1.43 (−1.57)
Ear and labyrinth disorders	169	0.45 (0.39–0.52)	0.45 (112.53)	0.45 (0.4)	−1.14 (−1.37)
Reproductive system and breast disorders	119	0.17 (0.14–0.2)	0.17 (497.22)	0.17 (0.14)	−2.58 (−2.84)
Congenital, familial and genetic disorders	78	0.29 (0.24–0.37)	0.29 (131.93)	0.3 (0.25)	−1.76 (−2.09)
Social circumstances	49	0.13 (0.1–0.17)	0.13 (287.56)	0.13 (0.1)	−2.95 (−3.35)
Product issues	34	0.02 (0.02–0.03)	0.02 (1328.42)	0.02 (0.02)	−5.34 (−5.82)
Pregnancy, puerperium and perinatal conditions	8	0.02 (0.01–0.04)	0.02 (357.61)	0.02 (0.01)	−5.54 (−6.5)

Abbreviations: PRR, Proportional Reporting Ratio; ROR, Reporting Odds Ratio; SOC, System Organ Class.

In addition to confirming these expected SOCs, our analysis revealed several potential SOCs not prominently highlighted in current prescribing information. Notably, nervous system disorders, although not meeting all four signal detection criteria, accounted for a substantial number of reports (*n* = 4606), suggesting a previously under‐recognized risk associated with the combination therapy. Other SOCs such as cardiac disorders (including myocarditis) and neoplasms benign, malignant and unspecified (including leukemia) also emerged as potential areas of concern, warranting further clinical attention.

**FIGURE 4:**
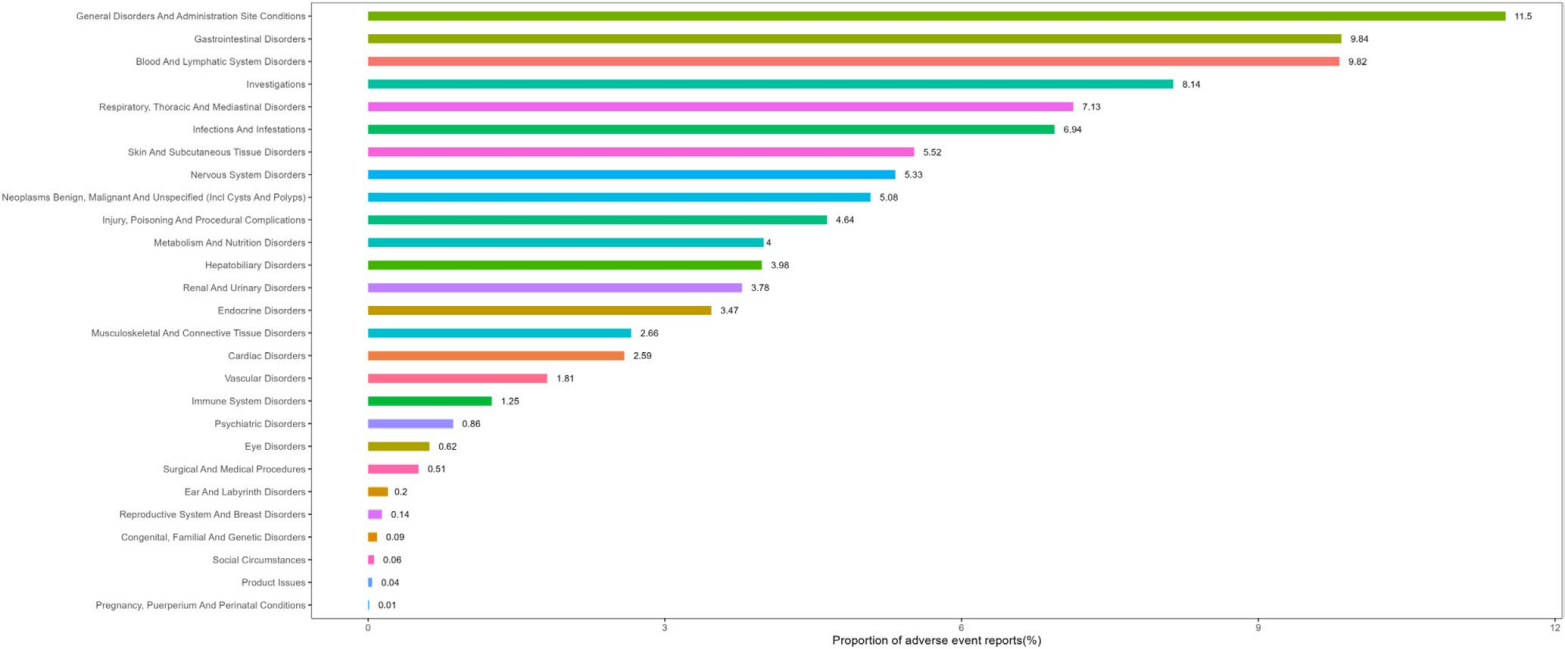
Proportional analysis of adverse events by System Organ Class, ordered by descending frequency, for the combination therapy of immune checkpoint inhibitors and platinum‐based compounds.

Figure 4 illustrates the proportional distribution of AEs across SOCs, further contextualizing the frequency and relative burden of these organ‐level toxicities.

### 3.3 Signal Detection Based on PT Levels

In the Preferred Term (PT) level analysis, we selected PTs meeting four predefined criteria after excluding events potentially associated with combination therapy of ICIs and platinum‐based compounds or clearly unrelated to pharmacologically‐induced adverse reactions. The remaining PTs were ranked by reporting frequency, with the top 50 PTs being presented (Table 2), while the complete list is provided in Table S3. Frequently reported adverse reactions included malignant neoplasm progression (ROR = 16, PRR = 15.63, EBGM = 15.27, IC = 3.93), anemia (ROR = 5.25, PRR = 5.18, EBGM = 5.14, IC = 2.36), and neutropenia (ROR = 7.2, PRR = 7.11, EBGM = 7.04, IC = 2.82). Notably, we also identified several rare PTs, such as neuropathy peripheral (ROR = 4.87, PRR = 4.84, EBGM = 4.81, IC = 2.27), leukemia (ROR = 4.49, PRR = 4.48, EBGM = 4.46, IC = 2.16), and myocarditis (ROR = 16.3, PRR = 16.26, EBGM = 15.87, IC = 3.99).

**TABLE 2:** Analysis of signal strength at the Preferred Term (PT) level for adverse events associated with combination therapy of immune checkpoint inhibitors and platinum‐based compounds in the FAERS database.

PT	a	ROR (95% Cl)	PRR (*χ* ^2^)	EBGM (EBGM05)	IC (IC025)
Malignant neoplasm progression	2148	16 (15.32–16.71)	15.63 (28741.15)	15.27 (14.73)	3.93 (3.87)
Anemia	1427	5.25 (4.98–5.53)	5.18 (4786.39)	5.14 (4.92)	2.36 (2.29)
Febrile neutropenia	1381	15.31 (14.51–16.16)	15.08 (17750.4)	14.75 (14.1)	3.88 (3.8)
Neutropenia	1367	7.2 (6.83–7.6)	7.11 (7107.74)	7.04 (6.73)	2.82 (2.74)
Pyrexia	1320	2.67 (2.53–2.82)	2.64 (1348.54)	2.63 (2.52)	1.4 (1.32)
Neutrophil count decreased	1050	19.06 (17.92–20.27)	18.84 (17234.58)	18.32 (17.4)	4.2 (4.1)
Pneumonia	1035	2.29 (2.15–2.43)	2.27 (739.61)	2.27 (2.16)	1.18 (1.09)
Thrombocytopenia	900	5.75 (5.38–6.14)	5.7 (3464.45)	5.66 (5.36)	2.5 (2.4)
Acute kidney injury	863	4.1 (3.83–4.38)	4.07 (1986.6)	4.05 (3.82)	2.02 (1.92)
Decreased appetite	840	2.6 (2.43–2.78)	2.58 (815.83)	2.58 (2.44)	1.37 (1.27)
Myelosuppression	772	23.78 (22.13–25.56)	23.58 (16093.95)	22.76 (21.43)	4.51 (4.4)
Interstitial lung disease	758	11.45 (10.65–12.3)	11.35 (7035.49)	11.17 (10.52)	3.48 (3.38)
Platelet count decreased	745	4.91 (4.57–5.28)	4.88 (2282.18)	4.85 (4.56)	2.28 (2.17)
Pancytopenia	731	9.47 (8.8–10.19)	9.4 (5410.77)	9.28 (8.72)	3.21 (3.11)
Hypothyroidism	661	15.19 (14.06–16.41)	15.08 (8491.15)	14.75 (13.83)	3.88 (3.77)
Pneumonitis	634	17.57 (16.24–19.02)	17.45 (9572.36)	17.01 (15.92)	4.09 (3.97)
Neuropathy peripheral	628	4.87 (4.5–5.27)	4.84 (1900.97)	4.81 (4.5)	2.27 (2.15)
Disease progression	613	3.7 (3.42–4.01)	3.68 (1192.23)	3.67 (3.43)	1.87 (1.76)
White blood cell count decreased	581	3.74 (3.45–4.06)	3.73 (1153.9)	3.71 (3.46)	1.89 (1.77)
Colitis	544	10.83 (9.95–11.8)	10.77 (4744.08)	10.61 (9.88)	3.41 (3.28)
Renal impairment	525	4.49 (4.12–4.89)	4.47 (1404.61)	4.44 (4.13)	2.15 (2.02)
General physical health deterioration	517	3.32 (3.04–3.62)	3.31 (828.5)	3.29 (3.06)	1.72 (1.59)
Sepsis	513	3.22 (2.95–3.51)	3.2 (775.57)	3.19 (2.97)	1.68 (1.55)
Adrenal insufficiency	384	25.33 (22.87–28.05)	25.22 (8588.8)	24.29 (22.29)	4.6 (4.45)
Immune‐mediated enterocolitis	380	119.35 (106.93–133.2)	118.83 (37346.65)	100.11 (91.32)	6.65 (6.49)
Hepatic function abnormal	362	7.04 (6.34–7.81)	7.01 (1846.31)	6.95 (6.37)	2.8 (2.64)
Immune‐mediated hepatic disorder	349	215.09 (190.45–242.91)	214.22 (55255.83)	160.06 (144.57)	7.32 (7.15)
Hypokalaemia	336	5.18 (4.65–5.77)	5.16 (1119.08)	5.13 (4.69)	2.36 (2.2)
Hyperthyroidism	327	16.56 (14.83–18.48)	16.5 (4640.72)	16.1 (14.69)	4.01 (3.85)
Leukopenia	319	4.49 (4.02–5.02)	4.48 (856.91)	4.46 (4.06)	2.16 (1.99)
Alanine aminotransferase increased	315	3.54 (3.16–3.95)	3.53 (567.68)	3.51 (3.2)	1.81 (1.65)
Pulmonary embolism	311	2.23 (2–2.5)	2.23 (210.62)	2.23 (2.03)	1.15 (0.99)
Pleural effusion	308	3.5 (3.13–3.91)	3.49 (544.17)	3.47 (3.16)	1.8 (1.63)
Hyponatraemia	299	3.71 (3.31–4.15)	3.7 (585.47)	3.68 (3.35)	1.88 (1.71)
Hepatitis	288	8.01 (7.13–9)	7.99 (1739.46)	7.9 (7.17)	2.98 (2.81)
Mucosal inflammation	280	7.69 (6.83–8.65)	7.67 (1604)	7.59 (6.87)	2.92 (2.75)
Septic shock	275	4.58 (4.06–5.15)	4.56 (760.28)	4.54 (4.11)	2.18 (2.01)
Tubulointerstitial nephritis	272	10.4 (9.23–11.73)	10.37 (2267.01)	10.22 (9.24)	3.35 (3.18)
Stomatitis	263	3.06 (2.71–3.46)	3.06 (362.45)	3.05 (2.75)	1.61 (1.43)
Neoplasm progression	255	4.85 (4.29–5.49)	4.84 (770.88)	4.81 (4.34)	2.27 (2.08)
Aspartate aminotransferase increased	249	3.22 (2.84–3.65)	3.22 (378.58)	3.2 (2.89)	1.68 (1.5)
Respiratory failure	249	2.37 (2.09–2.68)	2.36 (195.53)	2.36 (2.13)	1.24 (1.06)
Hepatic cytolysis	241	18.99 (16.7–21.59)	18.94 (3976.07)	18.42 (16.54)	4.2 (4.01)
Myocarditis	234	16.3 (14.31–18.56)	16.26 (3266.75)	15.87 (14.24)	3.99 (3.8)
Cytokine release syndrome	232	11.07 (9.72–12.61)	11.04 (2083.15)	10.87 (9.75)	3.44 (3.25)
Blood creatinine increased	218	2.28 (2–2.61)	2.28 (156.36)	2.28 (2.04)	1.19 (0.99)
Rash maculo‐papular	213	6.91 (6.03–7.91)	6.89 (1061.64)	6.83 (6.1)	2.77 (2.57)
Immune‐mediated lung diseasE	207	152.51 (131.01–177.54)	152.15 (25029.68)	122.71 (108.06)	6.94 (6.72)
Liver disorder	206	3.29 (2.87–3.77)	3.28 (325.43)	3.27 (2.92)	1.71 (1.51)
C‐reactive protein increased	196	3.8 (3.3–4.37)	3.79 (400.63)	3.77 (3.36)	1.92 (1.71)

### 3.4 Time‐to‐Onset Analysis

**FIGURE 5:**
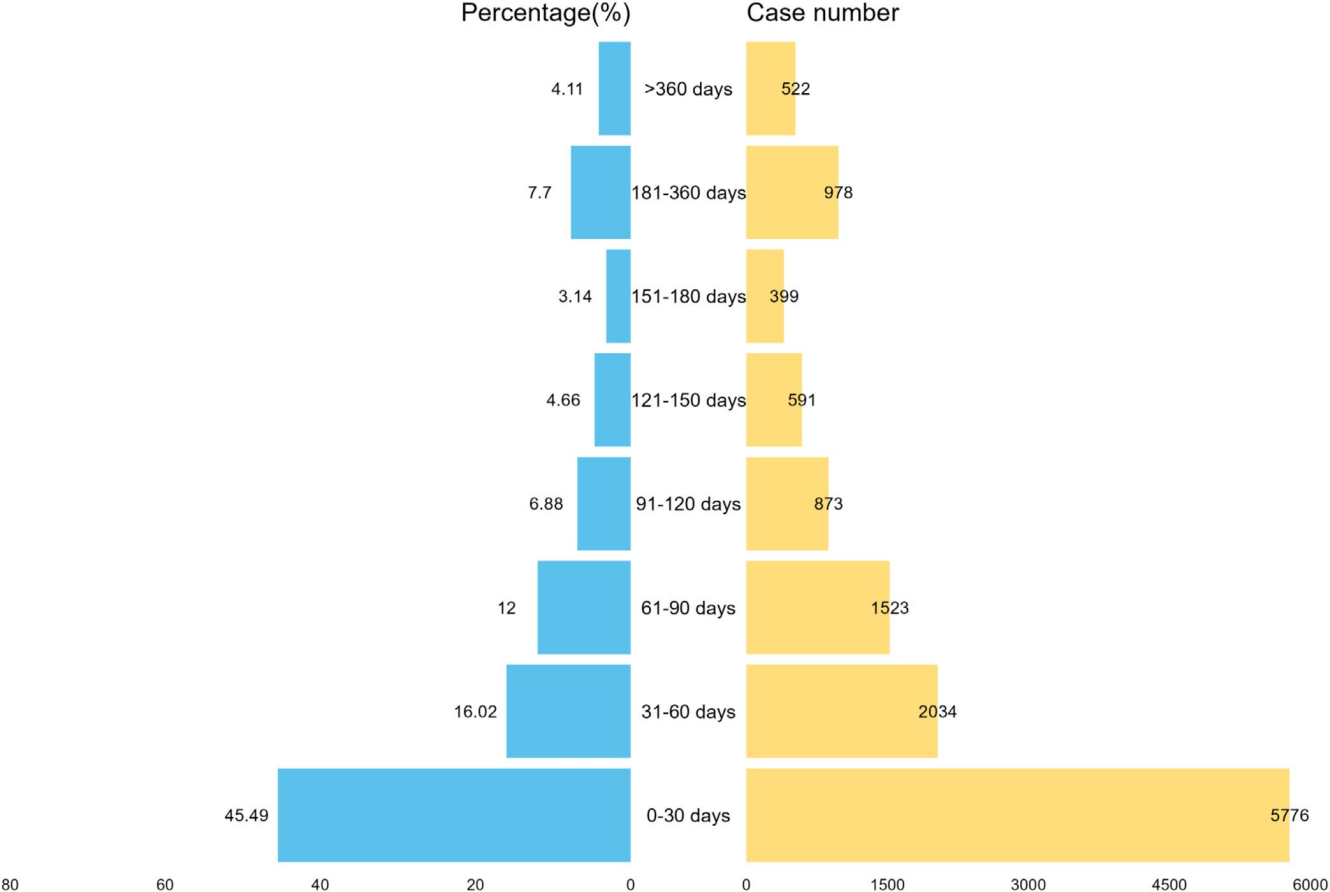
Onset time of adverse events associated with combination therapy of immune checkpoint inhibitors and platinum‐based compounds.

This study collected a total of 35,474 reports on the time to onset of AEs associated with ICIs combined with platinum‐based compounds from the database. After screening, which excluded duplicate entries, cases with incomplete demographic data (age or sex), and those lacking temporal information, 12,698 valid reports were ultimately included. The analysis revealed a median time to AE onset of 38 days (IQR: 12–96 days). As shown in Figure 5, 45.49% of cases (*n* = 5776) experienced AEs within the first month of combination therapy, indicating that the initial treatment phase represents a high‐risk period for AE occurrence, necessitating enhanced clinical monitoring.

### 3.5 Comparative Analysis

#### 3.5.1 Comparison Between Immune Checkpoint Inhibitor Monotherapy and Platinum‐Based Combination Therapy

Comparative safety analysis revealed a significantly lower incidence of treatment‐related AEs in the combination therapy group compared with the monotherapy group. Notably, the most pronounced differences were observed in the following AEs: malignant neoplasm progression (monotherapy, *n* = 17,687 vs. combination therapy, *n* = 2148), colitis (monotherapy, *n* = 3911 vs. combination therapy, *n* = 544), and pneumonitis (monotherapy, *n* = 3799 vs. combination therapy, *n* = 634). These findings suggest that the combination regimen may offer a more favorable safety profile while maintaining therapeutic efficacy. However, it should be noted that 628 cases of peripheral neuropathy were reported in the combination therapy group. Detailed data on AEs associated with ICI monotherapy are provided in Table S4.

#### 3.5.2 Comparison Between Platinum‐Based Agent Monotherapy and Immune Checkpoint Inhibitor Combination Therapy

The safety comparison demonstrated a significantly lower incidence of AEs in the combination therapy group compared with monotherapy. Notably, adverse reactions such as dyspnea (*n* = 5457) and vomiting (*n* = 4950) were observed in the monotherapy group but were not reported in the combination therapy group. Statistically significant reductions were also observed in the incidence of neutropenia (monotherapy *n* = 4577 vs. combination therapy *n* = 1367), thrombocytopenia (monotherapy *n* = 3879 vs. combination therapy *n* = 900), and acute kidney injury (monotherapy *n* = 2714 vs. combination therapy *n* = 863). These findings further indicate that the combination therapy may offer a superior safety profile. Relevant data for platinum‐based monotherapy are provided in Table S5.

### 3.6 Subgroup Analysis

#### 3.6.1 Gender‐Stratified Adverse Event Analysis

Tables S6 and S7 present the adverse reaction profiles of ICIs combined with platinum‐based compounds in male and female patients, respectively. The three types of AEs with the highest frequency were consistent across both sexes and included the following: malignant neoplasm progression (male *n* = 1201; female *n* = 800), febrile neutropenia (male *n* = 710; female *n* = 559), and anemia (male *n* = 670; female *n* = 644). Notably, interstitial lung disease demonstrated higher incidence in male patients (549 cases vs. 143 cases), while hepatitis occurred more frequently in female patients (167 cases vs. 100 cases).

#### 3.6.2 Age‐Stratified Adverse Event Analysis

Tables S8–S10 demonstrate the adverse event profiles of ICIs combined with platinum‐based agents across different age groups. The majority of adverse event reports originated from the elderly population (> 65 years), with malignant tumor progression (829 cases vs. 744 in younger adults) and anemia (673 cases vs. 550 in younger adults) being the most frequently reported. Although the number of reported cases was smaller in the adolescent group, the incidence of serious AEs—specifically delirium (26 cases) and mortality (5 cases)—was relatively high in this population. Notably, these events were not observed in middle‐aged and elderly patient groups.

## 4 Discussion

This study conducted a retrospective analysis of 28,585 reports from the FAERS database, systematically characterizing for the first time the adverse reaction profile of ICIs combined with platinum‐based agents. The results demonstrate that this combination regimen exhibits a distinct toxicity pattern, primarily manifested as significantly increased risks of early‐onset hematologic, endocrine, and hepatic toxicities, along with potential risks of neuropathy, myocarditis, and leukemia. These findings carry important implications for clinical practice.

The study identified 27 significant System Organ Class (SOC) signals, among which hematologic and lymphatic system disorders (ROR = 6.3, PRR = 5.78, EBGM = 5.73, IC = 2.52), endocrine disorders (ROR = 14.3, PRR = 13.84, EBGM = 13.57, IC = 3.76), and hepatobiliary diseases (ROR = 4.49, PRR = 4.36, EBGM = 4.33, IC = 2.12) met all four quantitative signal detection criteria. These findings partially overlapped with the known toxicity profiles of the individual drug classes: platinum‐based agents are well‐documented for causing myelosuppression and nephrotoxicity [18], while ICIs are prone to induce immune‐related endocrine disorders (e.g., thyroiditis) and hepatic injury [19]. Notably, the combination therapy markedly amplified hematologic toxicity risks (e.g., febrile neutropenia with ROR = 15.31), potentially attributable to platinum‐induced DNA damage activating the STING pathway to promote inflammatory cytokine release, which may synergize with ICIs to cause hematopoietic stem cell injury [20].

At the Preferred Term (PT) level, we identified three critical potential risks: (1) Peripheral neuropathy (*n* = 628, ROR = 4.87) emerged as a significant potential risk in the combination therapy group. The reporting frequency of this adverse event was significantly higher with combination therapy compared to ICI monotherapy. Previous literature indicates that neuropathies associated with ICI monotherapy are relatively uncommon [21], whereas the risk of neurotoxicity increases substantially when ICIs are combined with platinum‐based chemotherapy. The underlying mechanism may involve synergistic toxicity between the two drug classes: platinum‐based agents cause primary damage to neural tissues through direct cytotoxic effects, while ICIs systematically activate immune responses and create a pro‐inflammatory microenvironment, potentially exacerbating or triggering immune‐mediated nerve injury [22]. (2) Myocarditis (ROR = 16.3): Despite low incidence (*n* = 241), evidence suggests combination therapy increases myocarditis risk [23, 24]. The dual mechanistic hypothesis involves platinum‐induced cardiomyocyte damage releasing cardiac‐specific autoantigens (e.g., cTnI) and damage‐associated molecular patterns (DAMPs), while ICI‐driven T‐cell hyperactivation promotes effector T‐cell‐mediated attack on antigen‐expressing cardiomyocytes, exacerbating myocardial cell death [25]. (3) Leukemia risk (ROR = 4.49): This unexpected finding warrants cautious interpretation. No evidence suggests PD‐1/PD‐L1 inhibitors combined with cisplatin/carboplatin directly induces leukemia through dual mechanisms. Platinum agents exhibit genotoxicity potentially inducing mutations in hematologic malignancy‐associated genes (e.g., TP53, RUNX1) [26], whereas ICIs may promote clonal hematopoiesis through chronic immune activation [27, 28].

A total of 45.5% of AEs occurred during the first month of treatment (median time: 38 days), highlighting the importance of early monitoring. Hematologic toxicities (e.g., neutropenia) frequently developed 7–14 days after the first chemotherapy cycle [29], warranting weekly complete blood count monitoring in the initial month. Endocrine toxicities (such as thyroid dysfunction), though typically delayed in ICI therapy [30], exhibited early onset in this study, suggesting platinum‐based agents may accelerate autoimmune processes. Baseline and every 4‐week assessments of thyroid function and cortisol levels are recommended. Hepatotoxicity (e.g., immune‐mediated hepatitis, ROR = 215.09) requires close ALT/AST monitoring, particularly during the first 3 months of combination therapy [31].

Compared with monotherapy, combination therapy significantly reduced the incidence of certain toxicities: for malignant progression risk (combination group, *n* = 2148 vs. ICI monotherapy, *n* = 17,687), platinum compounds markedly enhanced the efficacy of ICIs by inducing immunogenic cell death (ICD) to release tumor antigens and remodel the tumor microenvironment (TME) [32]; for nephrotoxicity (acute kidney injury: combination group, *n* = 863 vs. platinum monotherapy, *n* = 2714), ICIs may mitigate platinum‐induced inflammatory renal injury by reversing T‐cell suppression and rebalancing the pro‐inflammatory environment [33]. However, the significant increase in neurotoxicity (combination group, *n* = 628) could adversely affect patients' quality of life and treatment adherence, warranting baseline nerve conduction studies for high‐risk populations (e.g., diabetic patients) along with prophylactic calcium/magnesium infusions (for oxaliplatin regimens) or gabapentin [34].

Gender differences were observed in toxicity profiles: males showed higher susceptibility to interstitial lung disease (*n* = 549 vs. 143 in females), while females exhibited greater hepatitis risk (*n* = 167 vs. 100 in males). This pattern aligns with sexual dimorphism in immune responses, where androgens may potentiate pulmonary Th17 reactivity whereas estrogens promote hepatic CD8+ T‐cell infiltration [35]. Age‐related risks demonstrated distinct patterns: adolescents displayed prominent delirium risk, likely attributable to blood–brain barrier immaturity facilitating enhanced central nervous system penetration of platinum agents [36]. Conversely, elderly patients (> 65 years) required particular vigilance against myelosuppression and infection risks.

This study represents the first large‐scale real‐world data analysis (*n* > 28,000) systematically comparing toxicity profiles between combination regimens and monotherapies. We employed multidimensional signal detection algorithms (ROR/PRR/BCPNN/MGPS) to enhance signal reliability. Several limitations inherent to the FAERS database should be considered in the interpretation of our findings. First, the voluntary nature of the reporting system introduces the potential for both underreporting and overreporting biases. While milder AEs are often underreported, more severe or clinically significant AEs may be disproportionately highlighted, leading to amplified signals for serious outcomes. Second, the absence of a definitive denominator representing the total exposed population precludes the calculation of incidence rates, thereby limiting our ability to quantify the absolute risk of specific AEs associated with ICI‐platinum combination therapy. Consequently, the associations identified in this study are statistical in nature and do not imply causality. Future prospective studies with well‐defined cohorts and adequate comparator arms are warranted to validate these findings and more accurately characterize the safety profile of these therapeutic regimens.

## 5 Conclusion

Through pharmacovigilance analysis of the FAERS database, we investigated the safety signals and potential risks associated with the combination of ICIs and platinum‐based compounds. The findings revealed that the combination therapy exhibits a distinct toxicity profile, primarily characterized by early‐onset hematological, endocrine, and hepatic toxicities. Compared with monotherapy, the combined regimen, while reducing the incidence of certain AEs (e.g., nephrotoxicity), introduced new risks such as neuropathy, myocarditis, and leukemia. Further research is warranted to elucidate the relationship between ICIs combined with platinum‐based drugs and these adverse reactions.

## Author Contributions

Conceptualization: Boyi Liu and Danna Chen. Data curation: Boyi Liu. Formal analysis: Boyi Liu, Ruizhe Huang, and Wenchao Zhang. Funding acquisition: Danna Chen. Investigation: Boyi Liu. Methodology: Boyi Liu and Wenchao Zhang. Software: Boyi Liu, Ruizhe Huang, and Dinwen Liu. Validation: Boyi Liu. Visualization: Boyi Liu, Jiaxing Liu, and Ao Han. Writing – original draft: Boyi Liu. Writing – review and editing: Boyi Liu, Yike Li, and Danna Chen. All the authors read and approved the final version.

## Funding

The authors declare that the research, writing, and publication of this article received funding. This study was jointly supported by the following grants: Hunan Provincial Department of Education Fund Project (23A0667); Changsha Outstanding Youth Innovation Talent Development Program (kq2106074); National Undergraduate Innovation and Entrepreneurship Training Program 2024 (Letter No. 13 [2024] from the Department of Higher Education—S202410823011); The “14th Five‐Year Plan” Applied Characteristic Discipline (Clinical Medicine) of Hunan Province; The 2024 Hunan Provincial Student Innovation and Entrepreneurship Training Program Project, Xiang‐ jiaotong [2024] 191 ‐ General Project ‐ 5276; Natural Science Founda‐ tion of Hunan Province (2026JJ81233).

## Conflicts of Interest

The authors declare no conflicts of interest.

## Supporting information


**Tables S1–S10:** cam471527‐sup‐0001‐TablesS1‐S10.xlsx.

## Data Availability

All original data can be accessed in the The FDA Adverse Event Reporting System (FAERS) database (https://open.fda.gov/data/downloads/).
